# Compact rover surveying and laser scanning for BIM development

**DOI:** 10.1371/journal.pone.0301273

**Published:** 2024-03-28

**Authors:** Syed Riaz un Nabi Jafri, Syed Murtaza Hussain, Asif Ahmed, Syed Asher Hussain Rizvi, Kumayl Hassan Kazmi, Jamshed Iqbal

**Affiliations:** 1 Department of Electronic Engineering, NED University of Engineering and Technology, Karachi, Pakistan; 2 Institute of Industrial Electronic Engineering-PCSIR, Affiliated with NED University of Engineering and Technology, Karachi, Pakistan; 3 Department of Computer Science and Technology, Faculty of Science and Engineering, University of Hull, Hull, United Kingdom; Al Mansour University College-Baghdad-Iraq, IRAQ

## Abstract

This paper presents a custom made small rover based surveying, mapping and building information modeling solution. Majority of the commercially available mobile surveying systems are larger in size which restricts their maneuverability in the targeted indoor vicinities. Furthermore their functional cost is unaffordable for low budget projects belonging to developing markets. Keeping in view these challenges, an economical indigenous rover based scanning and mapping system has developed using orthogonal integration of two low cost RPLidar A1 laser scanners. All the instrumentation of the rover has been interfaced with Robot Operating System (ROS) for online processing and recording of all sensorial data. The ROS based pose and map estimations of the rover have performed using Simultaneous Localization and Mapping (SLAM) technique. The perceived class 1 laser scans data belonging to distinct vicinities with variable reflective properties have been successfully tested and validated for required structural modeling. Systematically the recorded scans have been used in offline mode to generate the 3D point cloud map of the surveyed environment. Later the structural planes extraction from the point cloud data has been done using Random Sampling and Consensus (RANSAC) technique. Finally the 2D floor plan and 3D building model have been developed using point cloud processing in appropriate software. Multiple interiors of existing buildings and under construction indoor sites have been scanned, mapped and modelled as presented in this paper. In addition, the validation of the as-built models have been performed by comparing with the actual architecture design of the surveyed buildings. In comparison to available surveying solutions present in the local market, the developed system has been found faster, accurate and user friendly to produce more enhanced structural results with minute details.

## 1. Introduction

With the passage of time, the mobile robotics is innovatively providing various solutions to the industrial and domestic applications. The wheeled mobile robots are mostly holding the greatest shares among other possible robotic solutions [1]. The designing and fabrication of these robotic units are comparatively simple which leads to develop relatively easy strategies to control and navigate them in various environments to accomplish the assigned tasks [2]. The roboticists are utilizing the latest sensing and activation technologies in their developed moving robot and rover systems. Use of the modern laser scanners and vision sensors along with the standard proprioceptive sensors such as inertial measurement unit (IMU) has become the benchmark for modern rovers [3]. A variety of methodologies have been developed using various sensors to localize and navigate the rovers inside the targeted vicinities. Sooner it has realized that only rover’s localization is not sufficient to perform interactive tasks with in the environment until estimating the respective dimensions of the surrounding vicinities. Therefore the concept of Simultaneous Localization and Mapping (SLAM) has strongly evolved in the robotics research community and its various implementations have developed during past decades [4]. The researchers have utilized the SLAM technique to produce challenging industrial solutions using usual laser scan based point features along with true reflector land marks [5]. In domestic applications, the SLAM based mobile robots are more popular in multiple tasks such as educational, cleaning and other assistive applications. A research group produced an innovative solution to estimate the indoor emissions of CO_2_ using SLAM equipped rover navigation [6]. In order to improve the accuracy of the SLAM technique, the trend of fusing 2D and 3D laser scans with the sequential stereo and monocular camera images has become the favorite practice among the robotics researchers. Though the image information has boost the SLAM accuracies but still it is not fully apposite to large set of robotic platforms due to utilization of huge computational resources.

Meanwhile many interdisciplinary research works belonging to civil engineering domain were requiring the adoptability of modern autonomous navigational and mapping solutions for various surveying tasks. Therefore the stated sensor fusion practices have very broadly opened the doors of SLAM implementations in civil, construction and renovation engineering applications. The civil construction industry is probably one of the largest world’s industries having high number of labor involved with the availability of huge budgets. Since many decades, various construction related tasks such as inspection of built structures, assessment and management of undergoing projects, have performed through tedious, less accurate and time consuming manually operated tools and techniques. Advanced sensor technologies including stationary laser scanning systems started to replace the old instrumentations since the beginning of the new century [7]. However these systems were also requiring additional labor centric activities to place and calibrate the system at multiple desired locations inside the surveying vicinity to produce the complete solution. With the adoption of SLAM based mapping techniques in construction and surveying domain, role of semi-autonomous platforms and rovers has quickly increased since past few years [8]. The researchers combined the latest sensorial enhancements through SLAM methodology to provide the multiple times faster autonomous 2D and 3D mapping solutions as compared to traditional systems. Many research solutions have presented to the construction inspection community providing various semi-autonomous indoor mapping outcomes [9]. Though these solutions are quite accurate and less time consuming but on other hand demanding the significant financial resources to establish the required mapping hardware. Focusing on these challenges, a group of researchers introduced low-cost portable mobile laser scanning (MLS) method for 3D construction indoor mapping using IMU, Ultra-Wide Band (UWB) sensor and 2D laser scanner [10]. In addition to this, to further restrict the computational requirements of the mapping systems, various research works have utilized only 2D laser scans along with IMU feeds in SLAM framework to provide 3D robotic mapping of surveyed vicinities [11].

**Fig 1:**
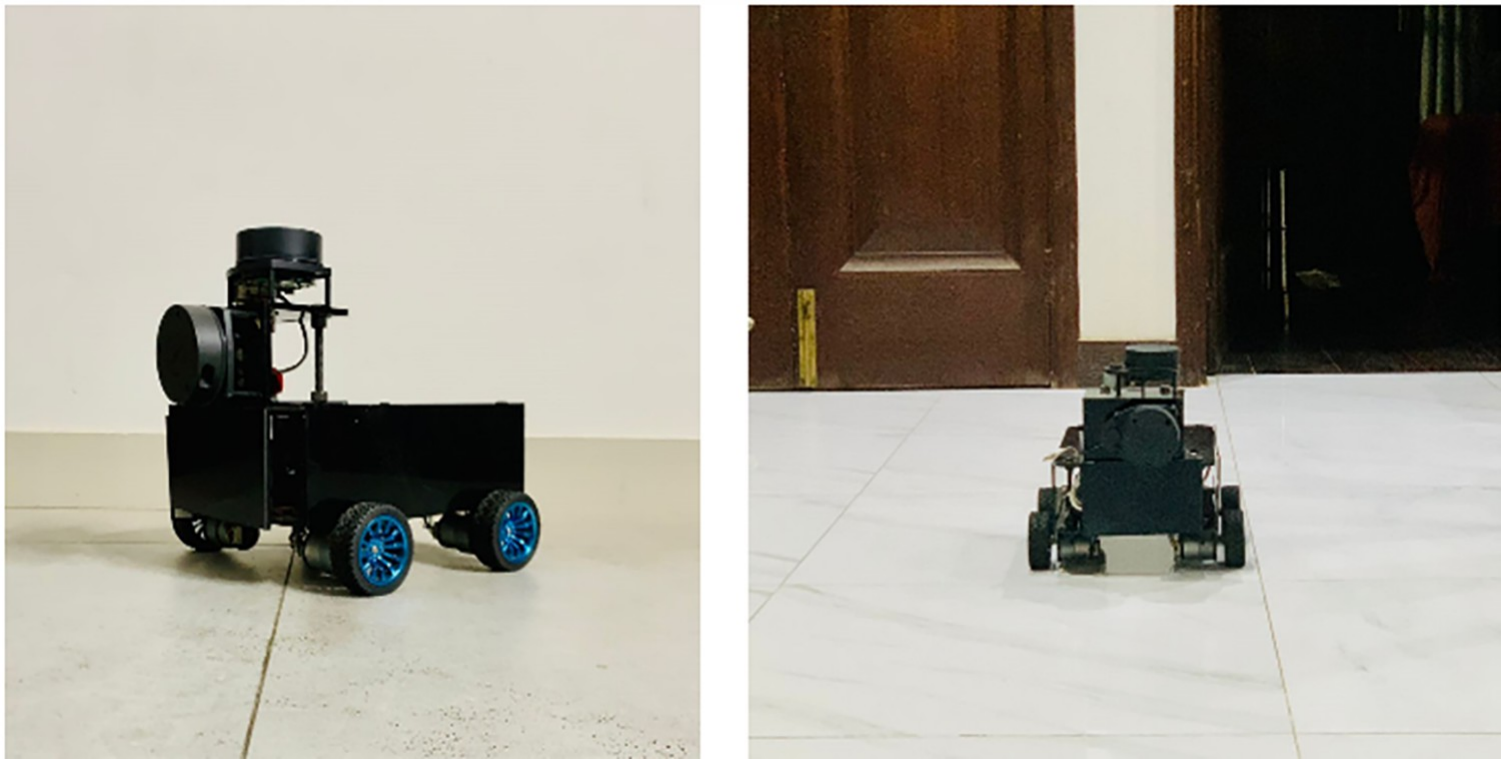
Custom made rover based 3D laser scanning and mapping system.

Due to the variations in the under construction surveying environments where large sets of distinct materials exists, the appropriate scanning and mapping tasks at affordable rate is quite challenging act that needs careful study and testing of existing laser scanners. Simultaneously, the movability of majority of existing mapping rovers is another vital challenge in partially occupied floors of surveying vicinities by keeping in mind the financial constraints of low budget projects. Therefore by continuing the stated research spirit and considering the requirements of the developing markets with tiny resourced projects, this research work is presenting the custom made highly economical compact rover based scanning and surveying solution for already built or under construction indoor environments as shown in Fig 1. The designing and fabrication of rover has been carried out using simple and low cost procedures to develop the moving system applicable for surveying and mapping of buildings and their surroundings. To restrict the cost of the system, affordable RPLidar A1series 2D https://instock.pk/slamtec-rplidar-a1-360-laser-range-scanner-development-kit-rplidar-a1m8.htmllaser scanners have selected and tested which offer satisfactory scanning outputs if compared price to performance wise with peers for low range operations [12].

**Fig 2:**
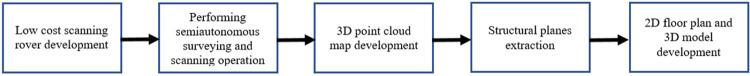
The block diagram of the overall mechanism of the 3D scanning and mapping system.

Globally many researchers already utilized the RPLidar scanners and provided quite acceptable mapping solutions on autonomous rovers at various indoor vicinities [13]. A group of researchers investigated the RPLidar based SLAM performance with UWB sensors and witnessed the good quality mapping outputs of the tested system [14]. In general, the usage of the RPLidar scanner in the state of art research works has restricted only to provide 2D mapping solutions. However the 2D mapping information is not quite descriptive for construction site inspections, therefore this research work is uniquely presenting the development of 3D mapping technique of the surveyed environment by integrating two RPLidar scanners orthogonally on the custom made rover. The overall mechanism of this research work is summarized in block diagram as shown in Fig 2. The developed rover is semi autonomously utilized to store the scans of the targeted region and by performing the offline computations on the perceived scans, the 3D point cloud map of the region is developed.

The 3D Cartesian point cloud mapping has been introduced since last decades to narrate the structural information of surveyed vicinities and now became a standard practice for various surveying solutions [15]. A group of researchers has successfully presented the technique to extract the roof bolts information from the developed 3D point cloud map [16]. In continuation to the applicability of the point cloud mapping, researchers have provided the building information modeling (BIM) solutions for distinct surveyed regions [17]. Similarly, this research work is providing the solution of 3D point cloud mapping and successfully utilizing the developed map to extract the structural planes and related information. Moreover the developed map is utilized to perform the building information modeling in order to produce the 2D floor plan and 3D structural model of the surveyed vicinities. Therefore a complete rover based scanning, mapping and BIM development solution is presented as summarized in Fig 2 for attainment of the local market needs.

In order to explain systematically the overall contributions of the research work, this paper is divided in multiple sections. Section 2 presents a brief discussion of the related research works. Section 3 demonstrates the mechanical and instrumentation design and fabrication of the scanning rover system. Section 4 explains the methodology of the point cloud generation, the structural plane extraction and the BIM development. While Section 5 elaborates the real experimental results of various indoor vicinities. Finally, the conclusions of the presented work have discussed.

## 2.  Related work

The earlier laser scanning based surveying systems were made using single laser scanner mounted on stationary platform. These kind of systems are very popular for surveying operations in a limited vicinity and a variety of products are commercially available with advanced sensing and computation facilities such as Leica HDS 7000 [18]. To survey broader vicinities in shorter time, the MLS systems have emerged and in use for various kind of indoor and outdoor challenging environments such as for forest inventory application [19]. Some companies have offered wearable backpack scanning systems such as LiBackpack MLS system for better surveying operations with additive sensor utilization [20]. In parallel to these surveyor held scanning systems, many researchers and companies did fascinating research investigations to enhance the quality of mapping by integrating laser scanners on stable wheeled moving platforms in order to smoothly conduct surveys and to get rich scanning point data. The TIMMS trolley based indoor mobile scanning and mapping system is one of the popular surveying solutions which provides dense and highly accurate mapping data in Global Positioning System (GPS) denies environments [21]. Enthusiastically the researchers belonging to robotics background, come up with fascinating autonomous robotic mapping solutions in order to ease the surveying jobs. An extraordinary mapping solution has produced by researchers using dog like SPOT robot carrying various 3D laser scanners to map the navigated vicinity [22]. Despite the marvelous performance of such complex robotic mapping systems, their affordability and operational requirements limit the usage globally.

**Fig 3:**
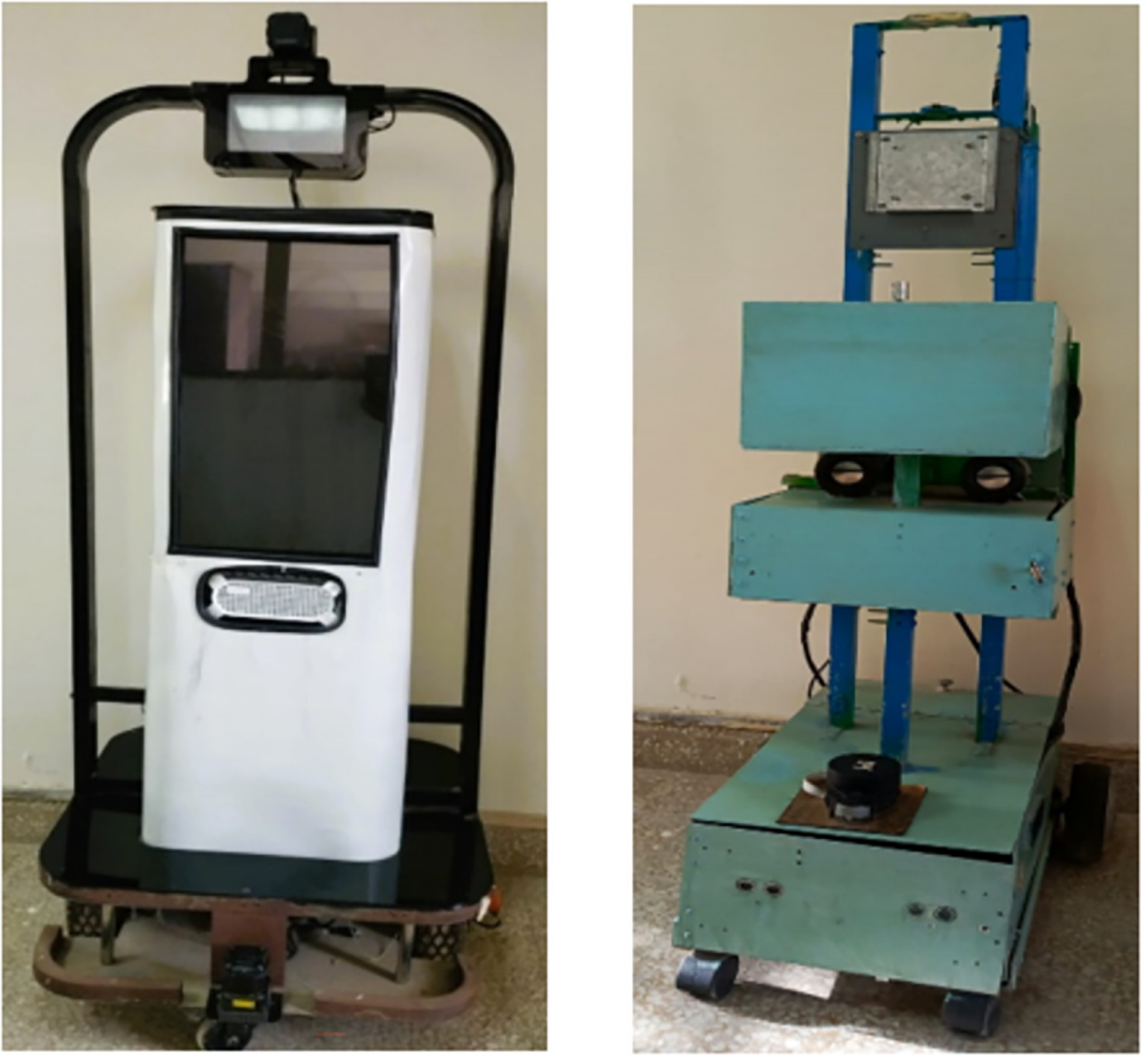
The robotic mapping platforms (left) an assistive mobile robot equipped with 2D Hokuyo laser scanners (right) an industrial mobile robot equipped with 2D RPLidar laser scanner.

However robotic developers consistently innovated various wheeled robotic platforms for surveying and mapping applications in order to ease the working procedures. Some researchers presented a mapping solution using autonomous unmanned ground vehicle (UGV) equipped with multiple laser scanners in order to generate the structural maps of the explored vicinities [23]. A local assistive robot variant for mapping operations equipped with Hokuyo 2D laser scanners is shown in the left of Fig 3. A group of research and development companies have produced the latest innovative outdoor mapping solution using autonomous marine survey vehicle equipped with laser scanners and navigational sensors [24]. The developed system produced the exceptionally well mapping of the river crossing points along with the sea bed topography. A popular Husky UGV robotic system for indoor and outdoor mapping has commercially produced by renowned robotic research company [25]. For tools dispatch operation, an indigenous industrial mobile robot equipped with RPLidar laser scanner is shown in the right of Fig 3. Another unique commercially available robotic mapping solution, the Jackal UGV is utilized by researchers of different universities to explore the potential of autonomous surveying and mapping of the targeted vicinities [26].

Apart to these extremely sophisticated high priced robotic mapping platforms, many researchers combined low cost wheeled platforms with affordable navigational and mapping sensors to produce low budget autonomous mapping solutions. A recent research work has used the economical iRobot create 2 platform along with the RPLidar A2 laser scanner for indoor scanning, monitoring and control applications [27]. A robotic company has produced a customized iRobot create 3 based platform with integrated Intel depth camera sensor along with RPLidar laser scanner for mapping and visualization tasks [28]. Some of its technical details have summarized in Table 1 along with other mapping rovers.

**Table 1:** Specifications of selected robotic mapping platforms.

Robotic Platforms	Number of Laser Scanners	Other Sensors	Mapping Range
3D mapping UGV [23]	1 (2D) 1 (3D)	Odometric sensors	260 m
Husky UGV [25]	2 (2D)	Odometric sensors, IMU, GPS	60 m
Jackal UGV [26]	1 (2D) 1 (3D)	Odometric sensors	100 m
LoCoBot-Base-R1 [28]	1(2D)	Odometric sensors and Depth camera	12 m

Another compact ROSMaster X1 rover is commercially provided with depth vision and RPLidar scanner for mapping and inspection tasks [29]. However the presented state of art economical platforms offer only limited and less accurate 3D mapping solution which cannot be applicable for constructional surveying tasks.

In parallel to the existing varieties of the scanning and mapping solutions, many research groups focused to exploit the advantages of the generated mapping outcomes to derive the additive models and information packages for various industries. A research group proposed a semi-autonomous approach to develop 3D BIM of surveyed buildings using the pre-registered 3D point cloud data sets [30]. Some researchers generated the parametric structure modeling for the 3D reconstruction of indoor environments using MLS based 3D point clouds [31]. The presented mechanisms developed on dense less noisy point cloud data sets while another research work showed good results for 3D as built modelling by incorporating deep learning model using the connectivity relations to classify the incomplete point clouds [32]. A terrestrial scanning based large indoor 3D point cloud data set has efficiently labeled for BIM related applications [33].

On a conclusive note, all the presented fascinating research works have enhanced the quality of the laser scanning based surveying platforms and have produced the computationally efficient methods to transform the scans to BIM. However majority of the solutions require handsome financial resources with the availability of highly skilled labor which are unfortunately scarcely available in developing markets. This research work is an effort to encompass the desired requirements of surveying to BIM process as per the developing markets need and to produce highly affordable solution with acceptable technical specifications.

## 3.  Designing of low cost rover based mobile scanning system

The surveying tasks require a compact easy to move rover based scanning system with necessary onboard instrumentation. In the first phase of the designing of the system, selection of laser scanner and its placement on the custom made rover system has carried out. While in the second phase, the required onboard instrumentation along with necessary peripheral components have developed and presented in detail as shown in following sections.

### 3.1 Selection of laser scanner

Though many research works have highlighted the mapping applications using depth or stereo vision sensors but due to the nature of surveying jobs of under construction regions where lighting conditions are very poor, the selection of appropriate laser scanning device has emerged as the most vital step for developing the system. The authors have discussed the characteristics of compact, economical and low weight 2D laser scanners for indoor surveying applications [12]. Indoor mapping tasks require perception accuracy at significant scanning rates with low signal to noise ratio in order to efficiently model the surveyed vicinities using scanning rovers. Based on the comparative performance of available scanners for low budget rover applications, the RPLidar A1 series 2D laser scanner which lies in class 1 laser safety standards, has selected for this indoor mapping project. This scanner has a valid sensing range of twelve meter around 360 degree field of view and capable to deliver 360 range measurements in a single scan [34]. A typical indoor scan *S_rn_* of RPLidar A1 series laser scanner with 360 range measurements has described in Eq (1).


$$S_{rn}=\{R_{0},R_{1},\ldots .,R_{359}\}$$ (1)

**Fig 4:**
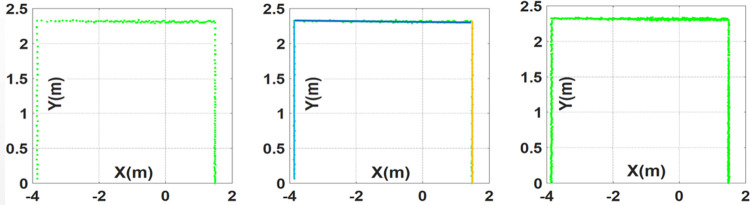
RPLidar scans (left) normal scan (middle) segmented scan (right) enhanced scan.

Here each *R_n_* is expressing the range measurement at particular angular displacement in degrees. By converting each range measurement into Cartesian space using relationships *x_n_* = *R_n_ Cosθ_n_* and *y_n_* = *R_n_ Sinθ_n_*, the Cartesian scan *S_cn_* can be described in Eq (2) and has shown in the left of Fig 4.


$$S_{cn}=\{\left(x_{0},y_{0}),(x_{1},y_{1}),\ldots .,(x_{359},y_{359}\right)\}$$ (2)

The respective scan *S_cn_* can be utilized online for 2D pose estimation and SLAM computations however for building 3D point cloud map, the scan size is quite small with scattered Cartesian points separated at one degree space. In order to increase the scan size with more Cartesian points at fraction of each degree space, the respective scan has processed offline using Split and Merge segmentation technique for finding the continuity of range measurements belonging to some regular surface [35]. The segmented scan has shown in the middle of Fig 4 with respective colored lines indicating various scanned planes. Each line segment is comprising of individual scan points separated at one degree space. The consecutive scan points have utilized to determine their mid-point using standard *x_mn_* = (*x_n_* + *x_n+1_*)/2 and y_mn_ = (*y_n_* + *y_n+1_*)/2 relationships. Further two mid points have determined in between (*x_n_,y_n_*), (*x_nm_,y_nm_*) and (*x_nm_,y_nm_*), (*x_n+1_,y_n+1_*) pairs respectively. Therefore three additional points have inserted in between each two consecutive scan points which are separated at angular step of 0.25 degree. It makes a greater scan size of 1437 points as described in Eq (3) and has shown in the right of Fig 4.


$$S_{cn}=\{\left(x_{0},y_{0}),(x_{0.25},y_{0.25}),(x_{0.5},y_{0.5}),(x_{0..75},y_{0.75}),(x_{1},y_{1}),\ldots .,(x_{358.75},y_{358.75}),(x_{359},y_{359}\right)\}$$ (3)

Those pair of scan points, which have not detected as part of any regular plane, hold the empty additional Cartesian points.

### 3.2 Mechanical design of scanning rover system

**Fig 5:**
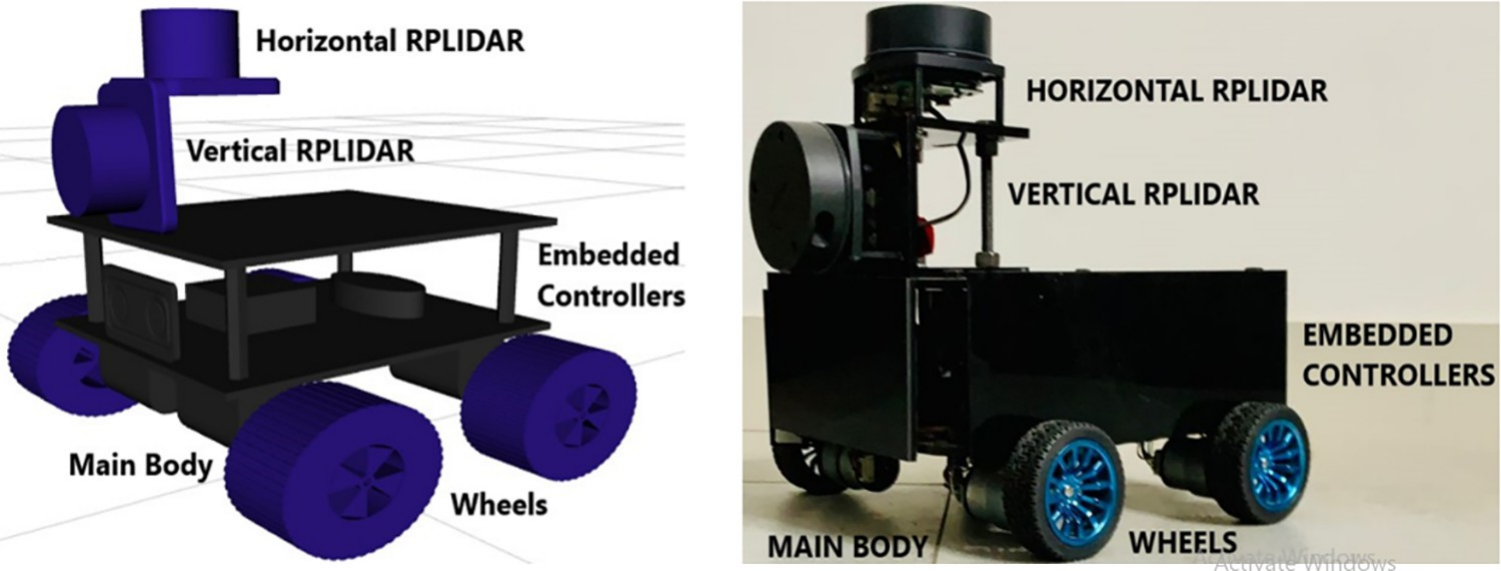
The custom made rover (left) CAD model (right) the fabricated system.

In order to develop the custom made economical rover based scanning system, at first step, the computer aided design (CAD) has constructed in Autodesk Fusion 360 software. The main rover system has designed using the base plate having mounting of four DC motors along with wheels. The top surface of the base plate has served as the housing for embedded controllers and peripheral electronics. A top plate has mounted on the base plate with a support of four small pillars and walls to cover the electronic components. An orthogonal L-shaped piece is specially connected on the top plate for the installation of two RPLidar A1 scanners in horizontal and vertical orientations. The complete labeled CAD model has shown in the left of Fig 5.

After designing the CAD model, the fabrication of the actual rover system has initiated. In order to keep the system compact, stable and low weight, the acrylic material has selected for construction of the rover. Two acrylic sheets have been cut in squared shape with a dimensions of 22x22 cm^2^. One sheet is sued as the base plate and four DC driving motors have installed on the bottom surface along with the four rubber tyre wheels. The wheels of 6 cm diameter are selected in order to sufficiently uplift the base plate from the floor. The two IR encoders have installed on front two wheels in order to get the rotational feedback of wheels. On the upper surface of the base plate, the batteries, power converter, Arduino controller, motion controller and Raspberry (Rasp) Pi embedded board have placed to run the rover as per commands. The second acrylic sheet has installed on the base sheet using 10 cm long four iron spacers. This sheet has used to cover the all electronics of base plate along with the walls and also served as the platform to interface two RPLidar laser scanners. An orthogonal acrylic L-shaped platform has developed for installing two laser scanners. On its top surface, the first RPLidar has connected horizontally to perceive the XY plane of the environment. On the front surface, the second RPLidar has connected vertically to perceive the YZ plane of the environment. The height of the L-shaped scanner’s platform is selected as 9 cm to perform the proper scanning from the moving rover. The completely labeled custom made rover system has shown in the right of Fig 5. The rover design has been kept extendable with the possibility of integrating any other additional sensors for future enhancements and as per user requirements.

### 3.3 Instrumentation design of the scanning system

The instrumentation of the scanning rover system is comprising of two sections, first is dealing with its movement control and second is related to the interfacing of the perception sensors. For movement control of the rover, the Arduino Mega controller is used to deliver the motion commands to the H-bridge motor drivers which in turn rotates the respective motor-wheel pair. The feedback IR encoders have used to sense the rotation rate of front two wheels and deliver the information to Arduino controller. Here the Arduino controller is acting as the middle man role, it takes the motion commands from Rasp-Pi 3B embedded controller and sends back the recent encoder feedback data. The Rasp-Pi controller is the central computing agent of the rover which is running Robot Operating System (ROS) to manage the required operations of the rover [36]. The ROS is a collection of huge set of open source multi-tasking packages which can use for various operations such as rover navigation and sensors data logging. The ROS has a unique feature to share the data and commands with in various computing machines which are independently running individual ROS. Using this extraordinary feature, the Rasp-Pi ROS is used to interact with the ROS running on the surveyor’s laptop through Wi-Fi communication network. In this work, the laptop ROS has served as the master computation unit while the Rasp-Pi ROS has served as the slave unit. The laptop ROS has used to generate the motion commands as per the surveyor’s need using either manual Tele-Op package or by the self-defined ROS package. The Rasp-Pi ROS is the subscriber of all ROS commands publishing at the laptop ROS and vice versa.

**Fig 6:**
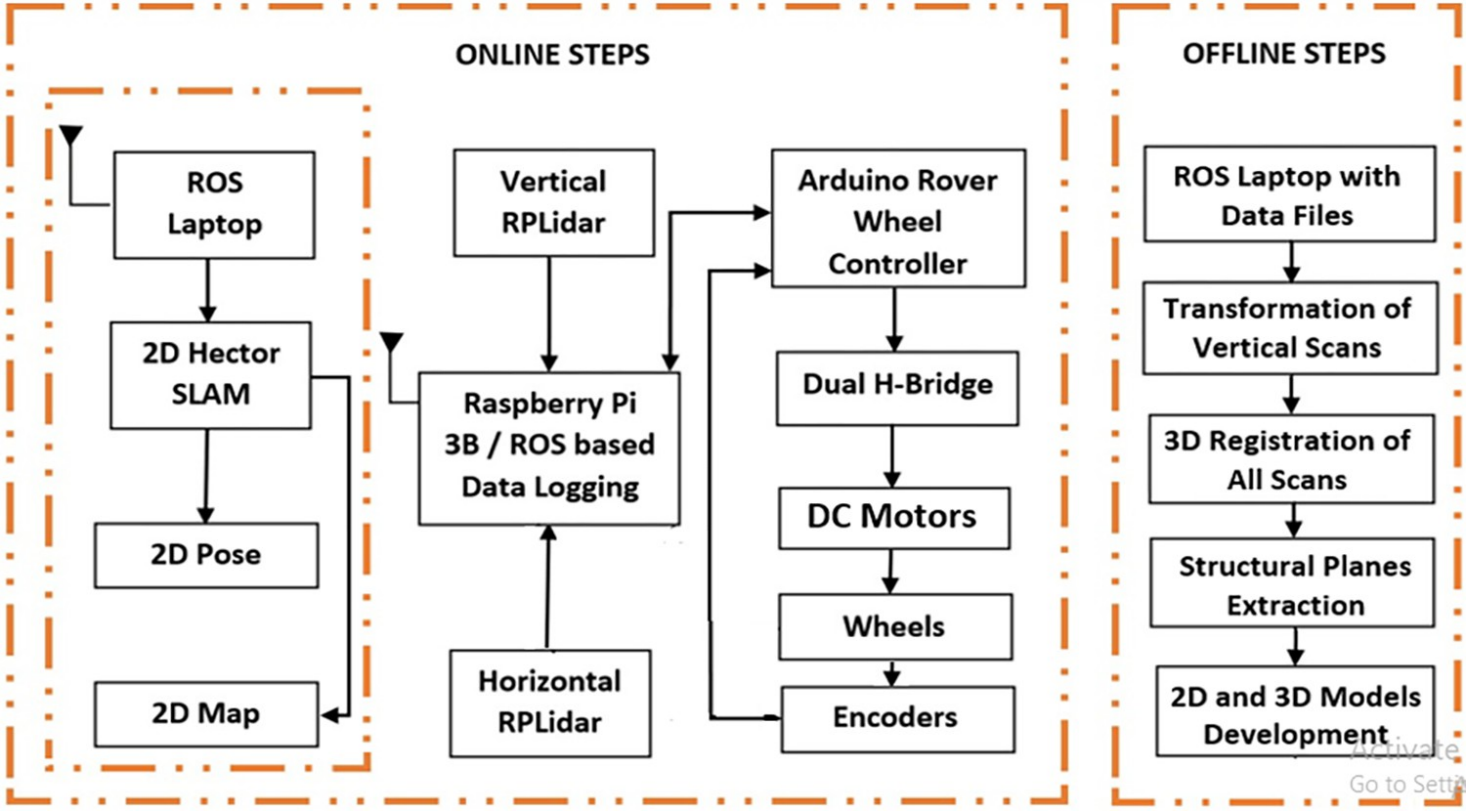
The block diagram of the instrumentation scheme.

For perception task of the environment, two RPLidar laser scanners have interfaced with the Rasp-Pi ROS. The horizontal and vertical RPLidars are used to provide environmental scans belonging to XY and YZ planes respectively. Both laser scans topics along with odometric encoder updates have subscribed by the laptop ROS for online visualization, processing and recording. Since the laptop ROS has more computational resources then Rasp-Pi ROS therefore some heavy computational packages have only processed on the laptop ROS such as the SLAM computation to generate the online poses of the moving rover along with the associated environmental 2D map. The complete instrumentation structure of the scanning system has shown in Fig 6 under the online steps section. Once the surveyor has completed the indoor scanning and data recording tasks through ROS based rover control than the offline processing on all sensorial recorded data has initiated to develop the 3D point cloud map and respective structural models as shown in Fig 6 under the offline steps section. The Matlab based registration of recorded scans of both scanners have carried out to transform and to develop the 3D point cloud map of the surveyed environment. The developed map is then further processed to extract the structural planes such as floor, walls and ceiling. In order to build the 2D floor plan and the 3D BIM of the surveyed building, the Autodesk Revit software is used to load the map and to construct the required models of the environment.

## 4.  Development of point cloud map and building information model

The surveying output of the custom made rover scanning system has made as the set of 3D environmental Cartesian points perceived during the controlled exploration of the targeted vicinity. The set of perceived Cartesian points is commonly referred as the 3D point cloud map of the environment. During the online scanning and recording of the sensorial data of the navigating rover, as a first step, only 2D pose estimation along with 2D map development have carried out using SLAM technique. The SLAM is used to estimate the most probable pose *x* and map *m* of the moving rover by factorizing the SLAM posterior *p(x_1:t_, m | z_1:t_, u_0:t−1_)* into factored form as shown in in Eq (4) [4]. In the expression, the 2D pose of the rover is denoted by *x*, the environment map by *m*, the horizontal laser scans by *z* and the odometric controls by *u*.


$$p(x_{1:t},m_{1:q}|z_{1:t},u_{0:t-1})=p(x_{1:t}|z_{1:t},u_{0:t-1}).\prod_{j=1}^{q}p(m_{j}|x_{1:t},z_{1:t})$$ (4)

**Fig 7:**
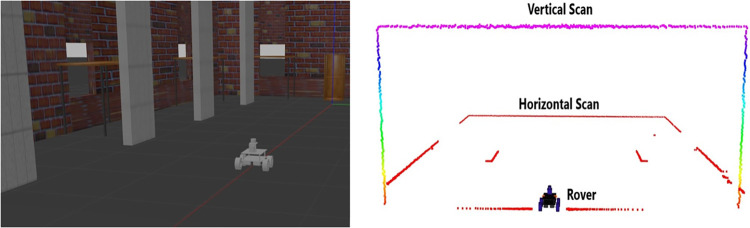
The simulated rover surveying (left) rover navigation inside simulated lab (right) visualization of scans.

There are various SLAM implementations available in ROS however the Hector SLAM ROS package has considered the optimal solution to generate the online 2D pose of the rover and the 2D environmental map [37]. The selected package offers a unique advantage of producing quite accurate pose estimation results even if encoder based odometric updates are too erroneous to use for the SLAM computations. In replacement to this, the package derives the odometry data through more stable scan matching technique by using consecutive scans and simultaneously provides the occupancy grid map of the environment. In order to see the surveying outcome using the selected SLAM package, a simulation test has carried out using the Gazebo ROS package. The similar rover model has developed in ROS using URDF scripting and a simulated lab like world environment in Gazebo has created containing walls, pillars and furniture as shown in the left of Fig 7. The rover has navigated in a straight path inside the lab using ROS Tele-Op commands where both the scanners have simultaneously scanned the vicinity and their visualization has shown in the right of Fig 7 using ROS RVIZ tool. Red and multi-color scan points belong to horizontal and vertical scans respectively.

**Fig 8:**
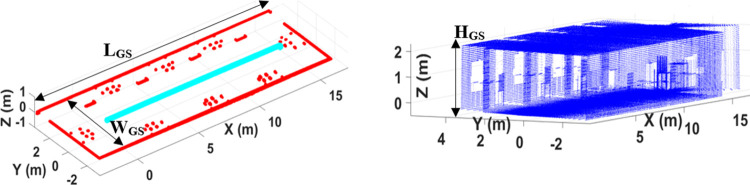
The mapping of the simulated lab (left) 2D pose and map of the lab (right) 3D point cloud map.

The online horizontal scans have utilized in Hector SLAM package to compute 2D pose and map of the environment as shown by red and cyan colors respectively in the left of Fig 8. The dimensions (*L_GS_*, *W_GS_*) of the 2D map have found accurate which indicated the correct performance of the Hector SLAM package. In order to develop the required 3D map of the environment, online ROS packages have not further utilized due to limited scan size of both scanners as discussed in earlier section. Therefore online recording of all scans along with estimated 2D poses have carried out using ROS bag feature for further offline processing. Later in the offline mode, the Matlab based custom made scripting has used to do various tasks in order to produce the 3D point cloud map. First the scan size of all recorded scans has increased and then each vertical scan has transformed to the local reference frame of the horizontal scanner. The vertical scanner has certain translational (*x_V_*, *y_V_*, *z_V_*) and rotational (*θ_VX_*, *θ_VY_*, *θ_VZ_*) displacements with respect to the horizontal scanner which are carefully set during the fabrication of the orthogonal platform. Using these displacements, every vertical scan point *P_V_* of each scan has transformed as the new scan point *P_VT_* with respect to the frame of reference of horizontal scanner using standard transformation procedure as shown in Eq (5).


$$P_{VT}=Trans(x_{V},y_{V},z_{V})Rot(Z,\theta_{Vz})Rot(Y,\theta_{Vy})P_{V}$$ (5)

All transformed scan points have formed new vertical scan *S_NT_* which has further combined with the horizontal scan *S_H_* to form the complete scan *S_F_* of the surveying system at any particular time as shown in Eq (6).


$$S_{F}=S_{H}+S_{NT}$$ (6)

Using the distinct recorded 2D poses (*x_Rq_*, *y_Rq_*, *θ_Rq_*) of the rover at particular time stamp *q*, the complete scan *S_F_* of the same time stamp has transformed to the global coordinated scan *S_Gq_* as shown in Eq (7).


$$S_{Gq}=Trans(x_{Rq},y_{Rq},0)Rot(Z,\theta_{Rq})S_{F}$$ (7)

All the distinct transformed scans of each particular time stamp have incrementally registered into the SLAM coordinated system to generate the consistent 3D point cloud map of the surveyed environment with a height of *H_GS_* as shown in the right of Fig 8.

**Fig 9:**
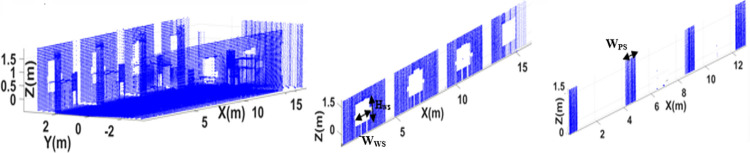
The extraction of structural planes (left) the point cloud map without ceiling (middle) wall (right) pillars.

The developed point cloud map contains various information aspects of the surveyed vicinity including the structural details along with the identification of furniture items. Therefore the extraction of structural planes have carried out from the point cloud map by modeling the standard plane equation “*ix+jy+kz = l*” using MATLAB implementation of Random Sample Consensus (RANSAC) algorithm [38]. Here each plane comprises of a set of Cartesian points, having a normal vector “*n_p_ = [i j k]”* and situated at a distance “*l”* from the origin. The ceiling plane is first extracted using *n_p_ = [0 0 1]* and temporarily removed from the point cloud map in order to see the clear interior view of the surveyed region as shown in the left of Fig 9. Later the left wall plane is extracted using *n_p_ = [1 0 0]* containing window openings as shown in the middle of Fig 9. During the development of the point cloud map, the transformed scan *P_VT_* contains the information of the height of the window *H_WS_*, while the complete registered scan *S_Gq_* contains the information of the width of the window *W_WS_*. Due to occlusion, there are always some discontinuities exist in point cloud map as observed by white spaces in the wall plane however they do not affect the overall extraction of respective plane. Each extracted plane has temporarily removed from the actual point cloud in order to extract other possible planes. Similarly another important structure of pillars have extracted *n_p_ = [1 0 0]* as shown in the right of Fig 9 with its dimensions labeled in the image.

**Fig 10:**
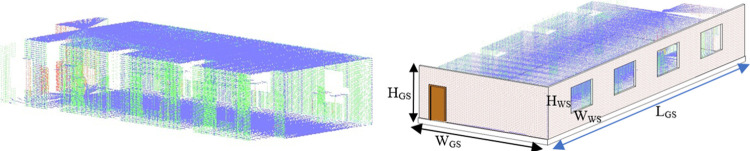
The processing of point cloud map (left) the point cloud map loaded in Revit software (right) the structural entities placement on point cloud map.

The point cloud map and the extracted structural information have used to develop the 2D floor plan and the 3D model of the surveyed environment for BIM development using Autodesk Revit software [39]. The point cloud map is loaded in the software as shown in the left of Fig 10. Using the already known dimensions of the point cloud map and the relevant structures, the placement of wall objects along with windows and doors have carried out on the point cloud map as shown in the right of Fig 10. Each object has assigned the same dimensions as observed during the development of the 3D point cloud mapping which indicates the efficacy of the overall surveying to BIM development process.

**Fig 11:**
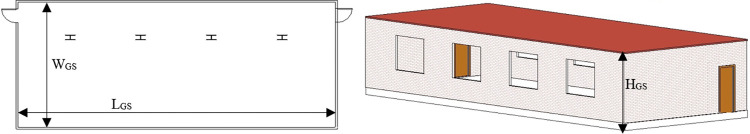
The BIM development (left) 2D floor plan of the lab (right) 3D model of the lab.

The developed 2D floor plan of the surveyed vicinity is shown in the left of Fig 11 with the same dimensions observed in the point cloud map. Finally the 3D model of the simulated lab is shown in the right of Fig 11 expressing the as built representation of the surveyed region. Using the same methodology, the surveying to BIM development has carried out for the real sites and presented in the next section.

## 5.  Surveying and scanning results of real sites

**Fig 12:**
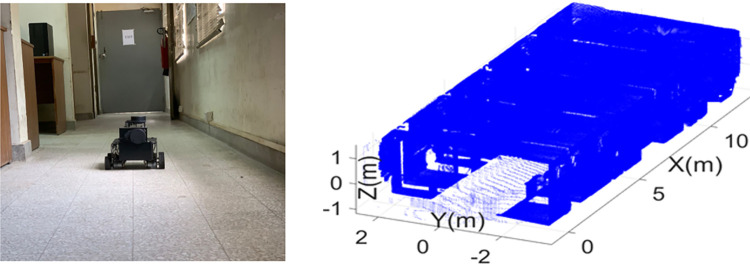
The mapping of the physical lab (left) the rover exploration inside the lab (right) the developed 3D point cloud map.

In order to survey the actual sites and to generate the BIM results using the presented scanning system, the interior of an academic building and a construction site have selected for testing the system. The first surveying task has carried out inside an academic lab of the building containing equipment and furniture as shown in the left of Fig 12. The rover has driven straight from one end of the lab towards other end for less than two minutes using ROS command. The online sensorial data has stored and processed offline to generate the 3D point cloud map of the lab as shown in the right of Fig 12.

**Fig 13:**
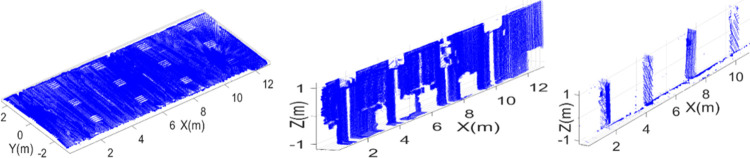
The extraction of structural planes (left) the ceiling (middle) the left wall (right) pillars.

As a first step, from the complete 3D point cloud map, main structural planes have extracted including ceiling, walls and pillars as shown in Fig 13. In ceiling plane, spots of light frames are visible as shown in the left of Fig 13, which shows the effectiveness and precision of surveying operation even for small objects present in the vicinity. The left wall plane has observed vacant spaces as shown in the middle of Fig 13, due to presence of other objects in front of the wall, such as tables, pillars or split AC units. The pillars have also extracted from the remaining point cloud as shown in the right of Fig 13.

**Fig 14:**
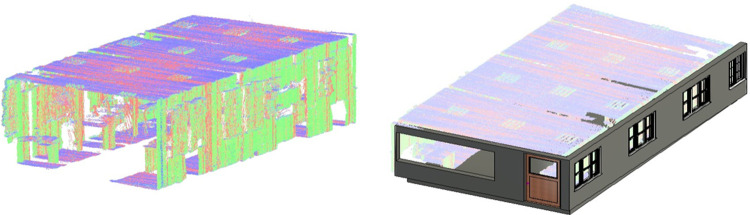
The processing of point cloud map (left) the point cloud map loaded in Revit software (right) the structural entities placement on point cloud map.

In the second step, the point cloud map has used to develop the 2D floor plan and the 3D model of the lab for BIM development. The point cloud map has loaded in the Autodesk Revit software as shown in the left of Fig 14. Using the known dimensions of the point cloud map and the relevant structures, the walls, windows and door objects have placed on the loaded map as shown in the right of Fig 14.

**Fig 15:**
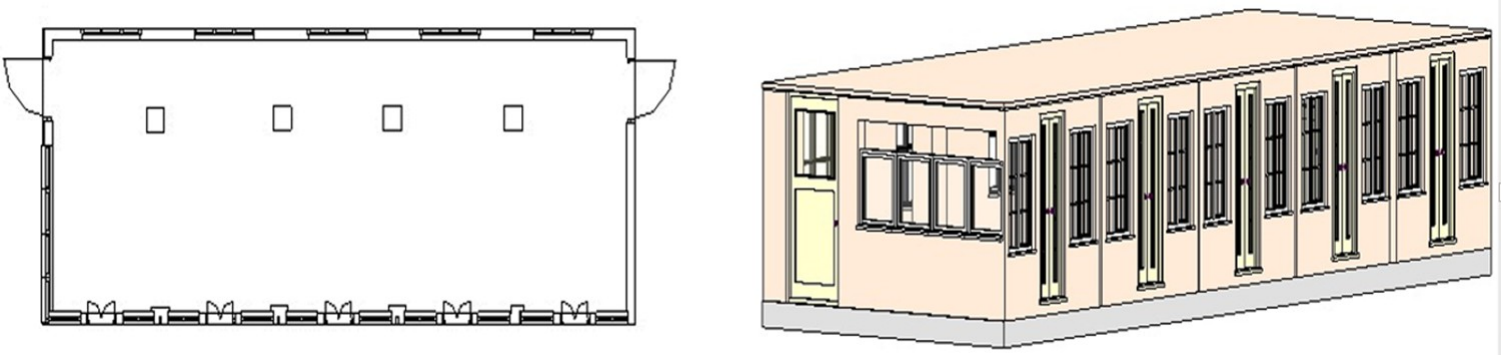
The BIM development (left) 2D floor plan of the lab (right) 3D model of the lab.

The developed 2D floor plan of the lab is shown in the left of Fig 15 with all structural features. The 3D model of the lab is shown in the right of Fig 15 which is clearly presenting the doors, windows and other structural planes.

**Fig 16:**
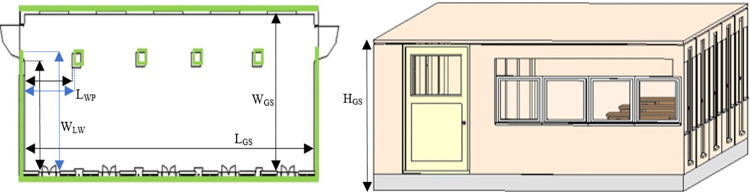
The BIM comparison (left) design to As-Built matching (right) enhanced 3D model of the lab.

The dimensions of the developed 2D floor plan has compared with the actual CAD values of the lab as shown in the left of Fig 16. The overall length *L_GS_*, width *W_GS_* and height *H_GS_* have found almost accurate as shown in Table 2. Very small absolute percentage errors have found w.r.t. the CAD values for these parameters. However few measured interior dimensions have mismatched with the actual design such as the left wall length *W_LW_* as presented in Table 2. It may be happened due to intentionally extending the door size of the lab during construction. Similarly the pillar displacement *L_WP_* from the wall has few cm difference if compared with the actual design. In general, the as-built information gathering through rover based scanning has found quite helpful to update the old design parameters and does not require highly sophisticated MLS platforms commonly utilized for various applications [31]. In addition, major furniture items have also added inside the 3D model of the lab as shown in the right of Fig 16. The overall rover scanning operation has repeated twice and results have compiled similarly as discussed earlier. No significant deviation has noticed to report when all independent scanning results have compared. This is indicating the coherence of the compact rover surveying mechanism over traditional counterparts.

**Table 2:** Comparison of the dimensions of the lab.

Parameters	Measured values (m)	CAD values (m)	Absolute error (%)
Length (*L_GS_*)	12.97	13.0	0.23
Width (*W_GS_*)	5.51	5.5	0.18
Height (*H_GS_*)	2.30	2.3	0
Left wall length (*W_LW_*)	4.10	4.2	2.38
Pillar displacement (*L_WP_*)	1.78	1.8	1.11

**Fig 17:**
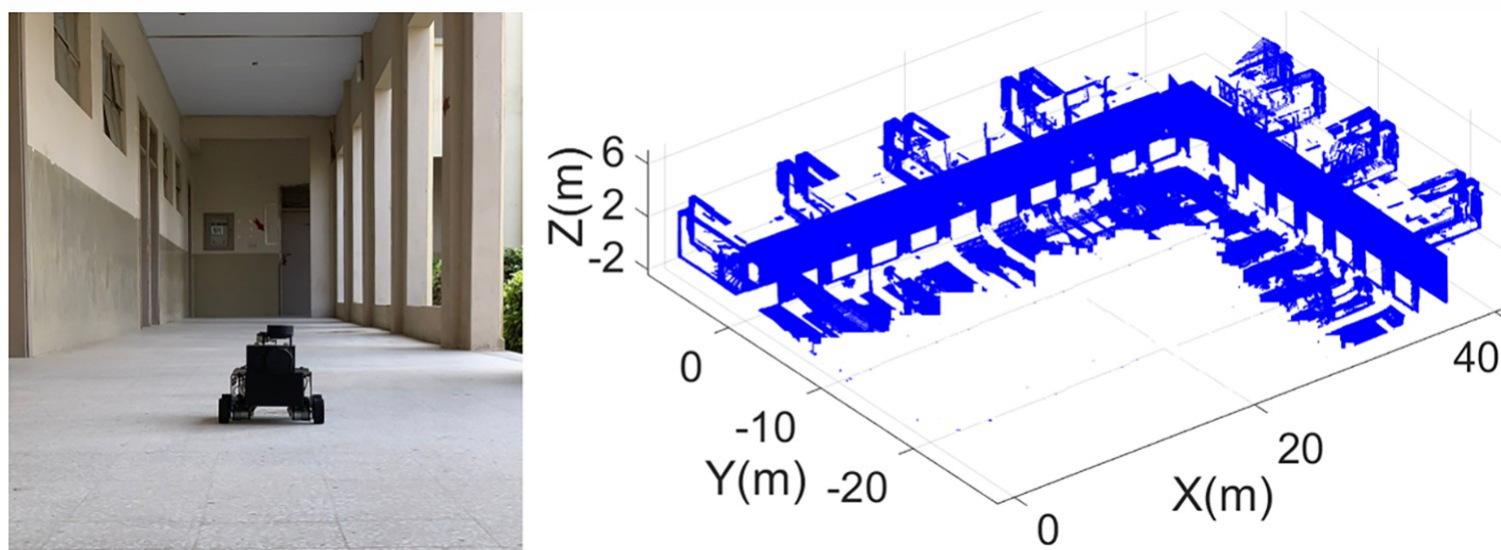
The corridor mapping (left) the rover exploration inside the corridor (right) the developed 3D point cloud map.

The second surveying job has carried out in an academic corridor by placing the rover at an end of the vicinity. The corridor has multiple class rooms and open spaces as shown in the left of Fig 17. The rover has driven in straight line and then turned right to move straight again till the end of the corridor. The overall scanning time was around six minutes and all the sensorial data was stored simultaneously. This is reflecting the greatest advantage of the rover based scanning operation as many other scanning mechanism such as terrestrial laser scanning requires long duration to completely scan larger vicinities [33]. Specifically, the local stationary scanning platform has taken ten times more duration for completing the scanning operation of the same corridor vicinity if compared with the presented rover based system [40]. In addition, the handling and performing surveying operation using compact rover has found much easier than the terrestrial scanning platforms and only one surveyor did the job quite easily. During the scanning operation, some erroneous pose estimation was observed after turning due to limited scan points of the vicinity and that error was manually corrected by taking few actual measurements. Later in the offline mode, the 3D point cloud map of the vicinity has generated as shown the right of Fig 17.

**Fig 18:**
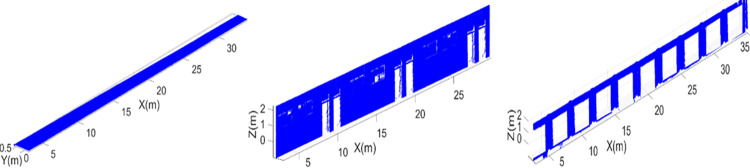
The extraction of structural planes (left) the ceiling (middle) left wall (right) right wall.

The developed point cloud map is quite dense containing multiple structural planes in the form of scanned Cartesian points. Using the same plane extraction scheme, the ceiling of the corridor has extracted as shown in the left of Fig 18. Similarly, the left wall plane has extracted from the remaining point cloud containing doors and windows as shown in the middle of Fig 18. Finally the right wall plane has extracted containing pillars and open spaces as shown in the right of Fig 18.

**Fig 19:**
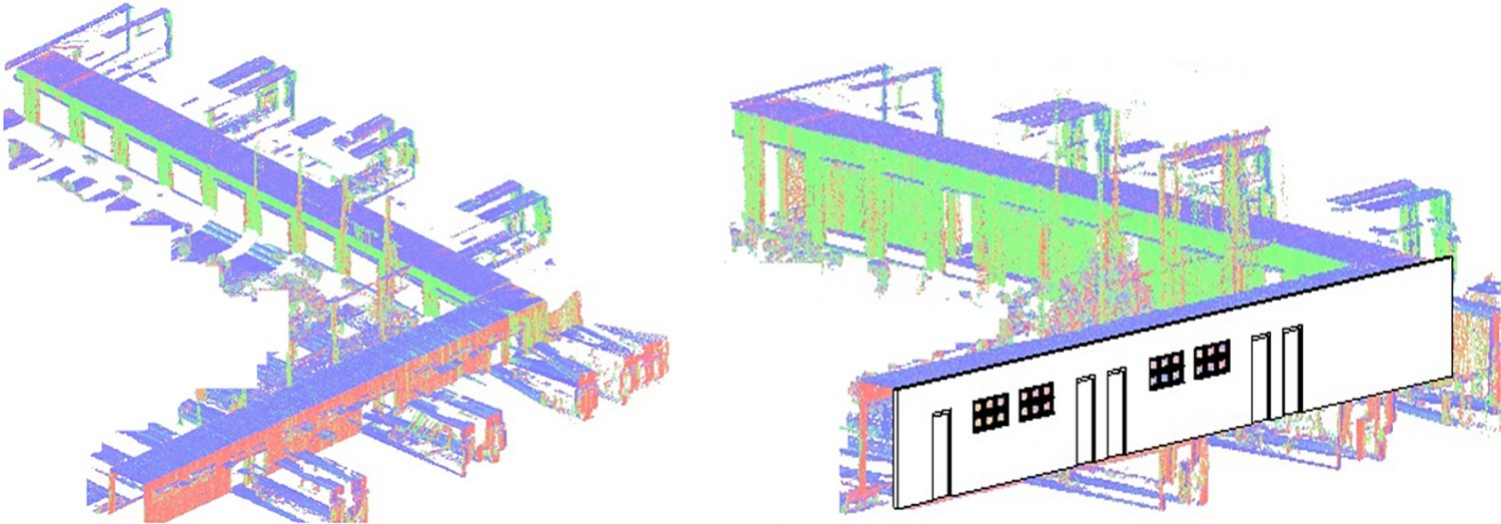
The processing of point cloud map (left) the point cloud map loaded in Revit software (right) the structural entities placement on point cloud map.

In continuation to this, the point cloud map has used to develop the 2D and 3D models of the corridor by loading it in the Autodesk Revit software as shown in the left of Fig 19. Using the dimensions of the point cloud map and its relevant structures, the doors, windows and walls objects have placed on the loaded map as shown in the right of Fig 19.

**Fig 20:**
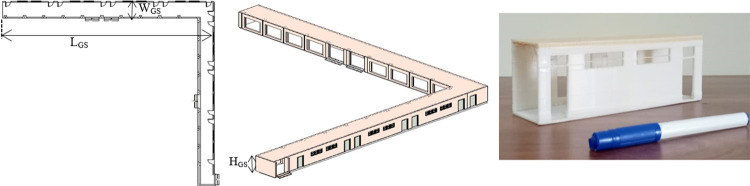
The BIM development (left) 2D floor plan of the corridor (middle) 3D model (right) 3D printed sample.

The final 2D floor plan of the lab is shown in the left of Fig 20 which is quite large then the previous surveyed regions and displaying walls, doors, windows and open spaces. The complete 3D model is shown in the middle of Fig 20 presenting the structural planes with details. The dimensions of the 2D floor plan has compared with the ground truth as the CAD model was not available due to older construction. The specified length *L_GS_*, width *W_GS_* and height *H_GS_* have found almost accurate as shown in Table 3. This is indicating the efficacy of the rover surveying if compared with tedious manual measuring or stationary scanning systems. At this stage, another attractive step has taken as per customer’s perspective by taking the 3D print of a small portion of the corridor to demonstrate physically the as-built information of the surveyed vicinity. A hundred time reduced sample 3D model has printed as shown in the right of Fig 20 presenting the two class doors, windows and open spaces on the opposite side of the wall. This printing enhancement has attracted locally the surveying stakeholders to visualize and to physically demonstrate their renovation or reconstruction ideas in front of possible investors.

**Table 3:** Comparison of the dimensions of the corridor.

Parameters	Measured values (m)	Ground truth (m)	Absolute error (%)
Length (*L_GS_*)	36.88	37.0	0.32
Width (*W_GS_*)	2.40	2.4	0
Height (*H_GS_*)	3.79	3.8	0.26

**Fig 21:**
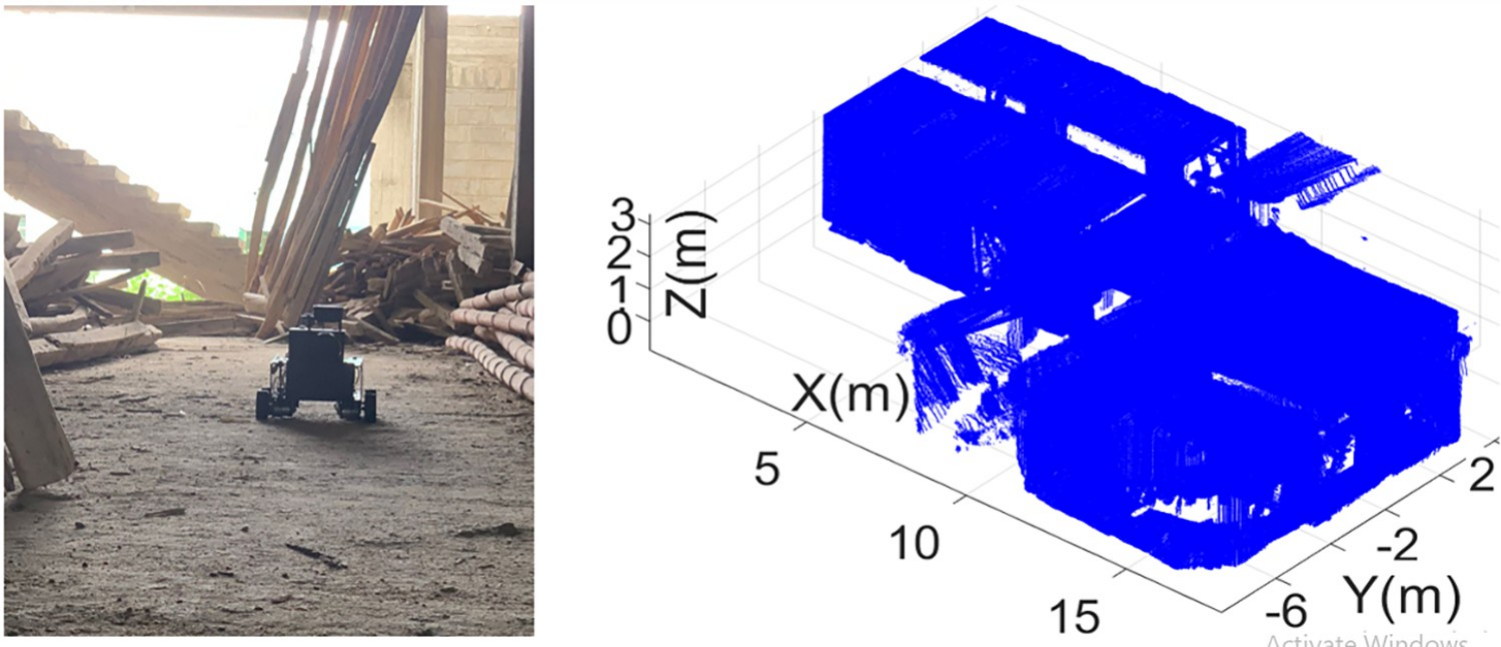
The mapping of the construction site (left) the rover exploration inside the vicinity (right) the developed 3D point cloud map.

The last surveying job has carried out inside a construction site by placing the rover at the starting location of the vicinity as shown in the left of Fig 21. This surveying test was very challenging due to presence of various construction material and the surveyor has chosen the most appropriate path for rover navigation. The rover has covered a significant distance inside the under construction vicinity in four minutes and all the sensorial data was stored in online mode. Later in the offline mode, the 3D point cloud map of the construction site has generated as shown the right of Fig 21. The point cloud map has many vacant openings due to presence of various tools and materials in disorder which partially blocked the scanning of relevant structural planes.

**Fig 22:**
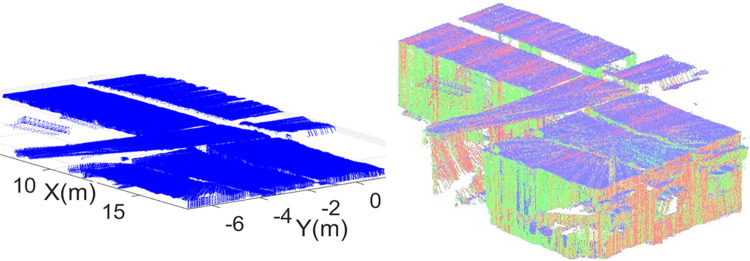
The processing of point cloud map (left) the ceiling plane extraction (right) the loading of point cloud map in Revit.

Using the developed 3D point cloud map, the ceiling plane has extracted as shown in the left of Fig 22 along with other structural planes. Later the point cloud map has loaded in the Autodesk Revit software as shown in the right of Fig 22.

**Fig 23:**
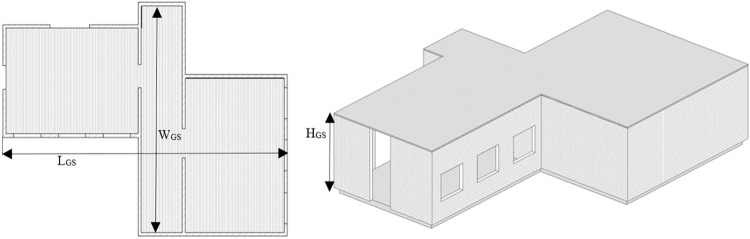
The BIM development (left) 2D floor plan (right) 3D model.

The 2D floor plan of the region is shown in the left of Fig 23 while the 3D model is finally generated as shown in the right of Fig 23. During the development of 3D model, surveyor’s visual aid has utilized to fill the vacant spaces present in the 3D map by wall or window objects. The physical dimensions of length *L_GS_*, width *W_GS_* and height *H_GS_* of the 2D floor plan has compared with the CAD model and found very nearer to the design parameters as shown in Table 4.

**Table 4:** Comparison of the dimensions of the construction site.

Parameters	Measured values (m)	CAD values (m)	Absolute error (%)
Length (*L_GS_*)	17.8	18.0	1.1
Width (*W_GS_*)	12.1	12.0	0.8
Height (*H_GS_*)	3.8	3.8	0

In general, the scanning rover system has easily utilized to develop as-built information of already developed vicinities while for under construction sites, it is observed that multiple scanning paths are required from different directions inside the targeted vicinity in order to completely perceive all structures which are occluded due to construction works. Therefore partially less accurate results have observed in construction site if compared with academic sites as mentioned in Tables 2–4 respectively.

## 6. Conclusions

This paper presented the development and working of the compact low cost rover based surveying and scanning system. The rover’s maneuverability has found quite smooth even at rough floors of the construction arena. The selected class 1 RPLidar laser scans have reflected back successfully within the acceptable signal to noise margins from distinct surfaces present inside the various surveying vicinities. The system has tested at various real sites and used to develop their 3D point cloud maps and building information models with minimal manual support. It is observed that the developed results are quite accurate if compared with the actual design of the surveyed vicinities and the measurement error has not exceeded to 2% of the ground truths. In addition, the surveying using laser scans has found quite descriptive in the sense that even the measurements of small stocks such as ceiling light frames have observed very accurate and can be used for future renovation tasks. Moreover few dimension corrections have also done to the actual design as observed during the rover scanning operation of the as-built structures. Apart to the accuracy of the results, the overall surveying operation has observed quite simple as the surveyor only needs to handle small rover along with the communicating laptop and the time consumption has very drastically reduced to 90% if compared with the existing surveying techniques of the developing markets. In addition the overall surveying system cost is affordable to the middle and low budget enterprises for accomplishing their projects with greater quality. Furthermore the presented BIM solution is reproducible in terms of 3D printing to generate the scaled version of the surveyed environment in order to concretely represent the new ideas in front of possible clients. The overall work is very helpful for various engineering disciplines including architectural to industrial engineering. As per some observations related to the system performance, it is considered to use better inertial and vision sensors to generate stable odometric updates and to use greater range laser scanners for enhancing the quality of the developed 3D point cloud map in future endeavors.

## Abbreviations

Acronyms SLAM: Simultaneous Localization And Mapping
ROS: Robot Operating System
BIM: Building Information Modeling
UGV: Unmanned Ground Vehicle
MLS: Mobile Laser Scanning
Variables and constants *R*_*n*_: N^th^ range value of respective scan mA
*S*_*rn*_, *S*_*cn*_: Single scan holding range values, single scan holding Cartesian point values NA
*x_n_, y_n_*: Nth Cartesian point of respective scan m
*P_VT_*: Transformed vertical scan point m
*x*_1:*t*_, *m*_1:*q*_: Pose and map values for respective samples NA
*z*_1:*t*_, *u*_0:*t*−1_: Measurements and odometric updates for respective samples NA
(*x*_*V*_, *y*_*V*_, *z*_*V*_): Translational displacements of vertical scanner w.r.t. horizontal scanner m
*θ_Vx_, θ_Vy_, θ_Vz_*: Rotational displacements of vertical scanner w.r.t. horizontal scanner rad
*S*_*H*_, *S*_*NT*_: Horizontal scan, new transformed vertical scan NA
*x_Rq_, y_Rq_, θ_Rq_*: Estimated 2D pose NA
*S_F_, S_Gq_*: Complete scan, globally transformed scan NA
